# Maximum-likelihood estimation of channel-dependent trial-to-trial variability of auditory evoked brain responses in MEG

**DOI:** 10.1186/1475-925X-13-75

**Published:** 2014-06-16

**Authors:** Cezary SieluŻycki, Paweł Kordowski

**Affiliations:** 1 Special Lab Non-invasive Brain Imaging, Leibniz Institute for Neurobiology, Brenneckestr. 6, 39118 Magdeburg, Germany; 2 Team Normal and Abnormal Motor Control, ICM Brain and Spine Institute, Sorbonne University, Pierre-and-Marie-Curie University (Paris 6), INSERM UMR1127, CNRS UMR7225, Hôpital Pitié Salpêtrière, 47 bd de l’Hôpital, 75013 Paris, France; 3 Department of Biomedical Physics, Institute of Experimental Physics, Faculty of Physics, University of Warsaw, ul. Hoża 69, 00-681 Warszawa, Poland

**Keywords:** Evoked responses, Habituation, Lateralization, Kronecker product, Maximum-likelihood estimation, MEG, Noise covariance, Trial-to-trial variability

## Abstract

**Background:**

We propose a mathematical model for multichannel assessment of the trial-to-trial variability of auditory evoked brain responses in magnetoencephalography (MEG).

**Methods:**

Following the work of de Munck *et al.*, our approach is based on the maximum likelihood estimation and involves an approximation of the spatio-temporal covariance of the contaminating background noise by means of the Kronecker product of its spatial and temporal covariance matrices. Extending the work of de Munck *et al.*, where the trial-to-trial variability of the responses was considered identical to all channels, we evaluate it for each individual channel.

**Results:**

Simulations with two equivalent current dipoles (ECDs) with different trial-to-trial variability, one seeded in each of the auditory cortices, were used to study the applicability of the proposed methodology on the sensor level and revealed spatial selectivity of the trial-to-trial estimates. In addition, we simulated a scenario with neighboring ECDs, to show limitations of the method. We also present an illustrative example of the application of this methodology to real MEG data taken from an auditory experimental paradigm, where we found hemispheric lateralization of the habituation effect to multiple stimulus presentation.

**Conclusions:**

The proposed algorithm is capable of reconstructing lateralization effects of the trial-to-trial variability of evoked responses, i.e. when an ECD of only one hemisphere habituates, whereas the activity of the other hemisphere is not subject to habituation. Hence, it may be a useful tool in paradigms that assume lateralization effects, like, e.g., those involving language processing.

## Background

Measurements of evoked brain responses in humans are an established standard in magneto- (MEG) and electroencephalography (EEG). The measured quantity is either the magnetic field above the surface of the head in MEG [1] or the electric field on the surface in EEG [2]. The sources of activity that underlie the measured signal are electric currents generated in a certain area of the cortex. Unfortunately, the background noise present in MEG and EEG measurements significantly exceeds the amplitude of the evoked brain activity of interest. The model commonly used to describe these data is the so-called signal-plus-noise (SPN) model. It assumes that: 1) the brain response to identical stimuli does not change from trial to trial, i.e. from one stimulation to another, and 2) the noise contaminating the response has a Gaussian distribution with zero mean. Hence, simple averaging of the sequentially recorded responses with respect to stimulus onset is supposed to yield a good estimate of the “real” brain response to the stimulus.

In the framework of the SPN model, the signal $R_{\text{ij}}^{\left(k\right)}$ measured on the *i*-th channel, at the *j*-th time moment, in the *k*-th trial, can be described as

(1) $$R_{\text{ij}}^{\left(k\right)}=R_{\text{ij}}+\varepsilon_{\text{ij}}^{\left(k\right)}\;,$$

where *i* = 1,…,*I*, *j* = 1,…,*J*, *k* = 1,…,*K*. *R*_*i* *j*_ is the brain response to the stimulus, which is assumed to be constant from trial to trial, and *ε* is a Gaussian noise with zero expected value, whose amplitude is typically larger than the amplitude of the evoked brain response. Due to the assumption of the Gaussian, zero-mean nature of the noise, *ε* is supposed to vanish on averaging across a large number of *R*^(*k*)^s.

Averaging across trials is useful when one is interested in the estimation of the evoked response, but in this approach information on the response variability across single trials is lost. In fact, the trial-to-trial variability is well documented by physiological studies. The analysis of single evoked responses has been addressed in various ways. For example, Bartnik *et al.* extracted and parametrized single evoked potentials in EEG by means of the wavelet transform [3]. Effern *et al.*[4], Quian Quiroga & van Luijtelaar [5], and Bagshaw & Warbrick [6] tried to increase the signal-to-noise ratio of single trials by wavelet denoising. Various statistical approaches—e.g., [7-9]—including new dedicated tests [10] were also proposed. De Munck *et al.* used a maximum-likelihood estimation [11], in which the spatial and temporal covariance of the contaminating noise was employed. Several coherence aspects were studied, for example, in [12-14]. Westerkamp & Aunon proposed a linear multielectrode filter to extract the single-trial responses [15], while Georgiadis *et al.* used the Kalman recursive filtering to obtain an estimate of a single-trial response by employing the information from the preceding trials [16]. Matching pursuit in the evaluation of the trial-to-trial variability of the auditory responses in MEG was used, e.g., in [17-20].

This paper aims at improving the quality of the estimators of auditory evoked brain responses by taking into account their trial-to-trial variability on the multichannel level. The current work is an extension of the work of de Munck *et al.*[11], where the spatio-temporal response pattern of each trial was weighted by a scalar coefficient assumed identical for all MEG channels. We propose to enhance that approach by estimating a weighting coefficient for each individual channel. This enables distinguishing between, e.g., habituation trends which differ for distinct groups of channels thus reflecting different trial-to-trial variabilities of the underlying sources—something which is not possible in the approach of de Munck *et al.*[11]. We apply the proposed method to simulated data as well as an illustrative real MEG data set from an auditory experiment, and discuss its limitations in the Discussion.

## Methods

### Assumptions

Based upon the realistic assumption that the trial-to-trial variation of evoked brain response should be taken into account when estimating the response amplitude, the following extension of the SPN model was proposed by de Munck *et al.*[11]:

(2) $$R_{\text{ij}}^{\left(k\right)}=\alpha^{\left(k\right)}R_{\text{ij}}+\varepsilon_{\text{ij}}^{\left(k\right)}\;,$$

where, for each trial *k*, *α*^(*k*)^ is a coefficient scaling the spatio-temporal amplitude pattern of the response, *R*, in this trial. In that work, the spatial and temporal covariance of the contaminating noise was estimated in order to minimize its influence on the estimates of *α* and *R*.

In this work, we propose to replace the scalar *α*^(*k*)^ with a diagonal matrix *ψ*^(*k*)^ in order to trace the trial-to-trial amplitude variation for individual channels *i* = 1,…,*I*:

(3) ψ(k)=ψ1(k)0…00ψ2(k)…0...................00…ψI(k).

Therefore, the order of the matrix *ψ*^(*k*)^ equals the number of channels *I*. In this way, in every single trial *k*, each of the channels *i* is assigned to the scalar coefficient corresponding to the evoked response amplitude variations on this channel. Thus, the fundamental equation of the considered process, (2), is replaced by

(4) $$R_{\text{ij}}^{\left(k\right)}=\psi_{i}^{\left(k\right)}R_{\text{ij}}+\varepsilon_{\text{ij}}^{\left(k\right)}\;.$$

Additionally, the following assumption is made

(5) ∑kψ(k)2=K0…00K…0.........00…K,

which, since *K* is the number of trials, says that in the case of non-variability of the response, each of the components on the leading diagonal of *ψ*^(*k*)^ is equal to one.

The model (4) poses further difficulties, because the dimensionality of the estimation process increases, yet we hope it offers a more accurate reflection of reality. Since it is known that the brain response evoked by a characteristic sensory stimulus arises in a particular area of the cortex (see, e.g., [21-24]), the magnetic fields measured by the channels corresponding to this area^a^ are stronger than those acquired from less active parts of the brain. Thus, it is reasonable to distinguish between individual channels when estimating the variability of the evoked response. For example, in their study of the habituation of the P300 wave for visual stimuli, Ravden & Polich observed that habituation was strongest at the Fz and Cz electrode sites [25].

Following the approach of de Munck *et al.*[26], the following additional assumptions concerning the noise space-time correlation are made: 

• the spatio-temporal covariance matrix, *C*, of the noise is the Kronecker product (see, e.g., [27]), ‘⊗’ of the spatial, *X*, and the temporal, *T*, covariance matrices,

(6) C=X⊗T,

• i.e., for $X_{ii^{\prime }}$ denoting the scalar being the spatial covariance between channel *i* and channel *i*^′^, and $T_{jj^{\prime }}$ being the temporal covariance between sample *j* and sample *j*^′^, *C* is of the following form 

• the noise of different trials is uncorrelated.

Although the dimensions of *C* = *X*⊗*T* are not smaller than the dimensions of the spatio-temporal covariance of the noise built traditionally, assumption (6) permits splitting *C* into *X* and *T*. Since the noise covariance matrix has to be estimated and inverted, this splitting is a clear advantage thanks to the fact that (*X*⊗*T*)^-1^=*X*^-1^⊗*T*^-1^.

It is clear from (6) that a common pattern of both *X* and *T* across trials *k* is assumed, as well as that *X* is time invariant and that *T* is space invariant, which of course is a simplification (see [28]), yet, a computationally efficient one.

### Estimation of evoked response varying across trials

To derive the maximum-likelihood estimators of *X*, *T*, *ψ*^(*k*)^ and *R*, one needs to find the distribution of $\varepsilon_{\text{ij}}^{\left(k\right)}$. Due to the assumption of the zero-mean, Gaussian nature of the noise and the aforementioned assumptions concerning the noise correlation, the covariance between two noise realizations (one on channel *i*, at sample *j*, in trial *k*, and the other on channel *i*^′^, at sample *j*^′^, in trial *k*^′^) equals [26]

(8) $$E\left(\varepsilon_{\text{ij}}^{\left(k\right)}\varepsilon_{i^{\prime }j^{\prime }}^{\left(k^{\prime }\right)}\right)=X_{ii^{\prime }}T_{jj^{\prime }}\;\delta_{kk^{\prime }}\;,$$

where $\delta_{kk^{\prime }}$ is the Kronecker delta defined as

(9) $$\delta_{kk^{\prime }}=\left\{\begin{array}{c} 1\;\text{for}\;\;k=k^{\prime }\\ 0\;\text{for}\;\;k\neq k^{\prime }. \end{array}\right)$$

Thus, the determinant of the overall covariance equals [27]

(10) $$\det\left(E\left(\varepsilon_{\text{ij}}^{\left(k\right)}\varepsilon_{i^{\prime }j^{\prime }}^{\left(k^{\prime }\right)}\right)\right)={\left(\det X\right)}^{\text{JK}}{\left(\det T\right)}^{\text{IK}}\;.$$

Moreover, since

(11) $$\begin{array}{c} \underset{i,j,k,i^{\prime },j^{\prime },k^{\prime }}{\sum }\left(R_{\text{ij}}^{\left(k\right)}-\psi_{i}^{\left(k\right)}R_{\text{ij}}\right){\left(X^{-1}\right)}_{ii^{\prime }}{\left(T^{-1}\right)}_{jj^{\prime }}\delta_{kk^{\prime }}\left(R_{i^{\prime }j^{\prime }}^{\left(k^{\prime }\right)}-\psi_{i^{\prime }}^{\left(k^{\prime }\right)}R_{i^{\prime }j^{\prime }}\right)=\\ \;=\underset{k}{\sum }\text{tr}\left(\left(R^{\left(k\right)}-\psi^{\left(k\right)}R\right)T^{-1}{\left(R^{\left(k\right)}-\psi^{\left(k\right)}R\right)}^{T}X^{-1}\right)\;, \end{array}$$

the probability density distribution *f*_*ε*_ of *ε* is expressed by

(12) $$\begin{array}{c} f_{\varepsilon }\left(R,\psi^{\left(k\right)},X,T\right)=\exp\left(-\frac{1}{2}\underset{k}{\sum }\text{tr}\left(\left(R^{\left(k\right)}-\psi^{\left(k\right)}R\right)T^{-1}{\left(R^{\left(k\right)}-\psi^{\left(k\right)}R\right)}^{T}X^{-1}\right)\right)\cdot \\ \;\cdot {\left(2\pi \right)}^{-\text{IJK}/2}{\left(\det T\right)}^{-\text{IK}/2}{\left(\det X\right)}^{-\text{JK}/2}\;. \end{array}$$

In the context of the maximum-likelihood estimation of *X*, *T*, *ψ*^(*k*)^ and *R*, (12) represents the likelihood function. For the sake of keeping the formulas simple, all estimators are denoted as non-dashed (i.e. by *X* instead of $\hat{X}$, for example).

### Calculation of the estimates of *X*, *T*, *ψ* and *R*

Differentiating (12) with respect to *X*, *T*, *ψ*^(*k*)^ and *R*, and setting the obtained derivatives to zero, one obtains the following estimators (see Appendix: Detailed derivations for detailed derivations).

(13) $$\begin{array}{l} X=\frac{1}{\text{JK}}\underset{k}{\sum }\left(R^{\left(k\right)}-\psi^{\left(k\right)}R\right)T^{-1}{\left(R^{\left(k\right)}-\psi^{\left(k\right)}R\right)}^{T},\; \end{array}$$

(14) $$\begin{array}{l} T=\frac{1}{\text{IK}}\underset{k}{\sum }{\left(R^{\left(k\right)}-\psi^{\left(k\right)}R\right)}^{T}X^{-1}\left(R^{\left(k\right)}-\psi^{\left(k\right)}R\right),\; \end{array}$$

(15) $$\begin{array}{l} \psi^{\left(k\right)}=\text{dg}\left({\left(X^{-1}⊙\left(RT^{-1}R^{T}\right)\right)}^{-1}\text{diag}\;\left(X^{-1}R^{\left(k\right)}T^{-1}R^{T}\right)\right),\; \end{array}$$

(16) $$\begin{array}{l} R={\left(\underset{k}{\sum }\psi^{\left(k\right)}X^{-1}\psi^{\left(k\right)}\right)}^{-1}\underset{k}{\sum }\psi^{\left(k\right)}X^{-1}R^{\left(k\right)},\; \end{array}$$

where ⊙ denotes the Hadamard product, i.e. (*Y*⊙*Z*)_*m* *n*_ = *Y*_*m* *n*_*Z*_*m* *n*_, diag denotes the operator resulting in a vector that contains elements of the leading diagonal of the matrix that diag acts on, and dg denotes the operator resulting in a diagonal matrix whose leading diagonal contains elements of the leading diagonal of the matrix (or elements of the vector, as in this case) that dg acts on and zeros elsewhere.

The above system of matrix equations (13)–(16) has no closed form solution. Hence, in order to find the approximated estimates of *X*, *T*, *ψ* and *R*, we propose to solve it iteratively, starting with *X*, *T* and *ψ*^(*k*)^ being identity matrices, and with $R=K^{-1}\sum_{k}R^{\left(k\right)}$. In such an approach, consecutive updates of *X*, *T*, *ψ* and *R* are computed based on their values from the previous iterations in which formulae (13)–(16) are computed consecutively. At the end of each iteration, each *ψ*_*i*_ is scaled according to (5).

### Material

Real MEG data were acquired from a healthy male volunteer (age 42, normal hearing), who is a member of the Special Lab Non-invasive Brain Imaging. The subject was exposed binaurally to 500-ms long 1-kHz sine tones at 90 dB SPL. The difference between the onsets of the consecutive stimuli was 2 s. The data were sampled at the frequency of 1017.25 Hz and filtered between 0.5 and 400 Hz. 160 trials were extracted out of 224 as artifact free. They were offset corrected for each channel with respect to the mean magnetic field over the 200-ms long prestimulus baseline time window.

## Results

### Simulations

In order to test to which extent the proposed methodology correctly assesses the channel-dependent trial-to-trial variability of the spatio-temporal response pattern *R*, we simulated two equivalent current dipoles (ECDs), one in each auditory cortex, whose time courses were habituating differently over trials. On the sensor level, the simulated signal can then be expressed as

(17) $$R_{\text{ij}}^{\left(k\right)}=L_{1}(i)h_{1}(k)s_{1}(j)+L_{2}(i)h_{2}(k)s_{2}(j)+\varepsilon_{\text{ij}}^{\left(k\right)}\;,$$

where *L*_1_(*i*) denotes the leadfield of source 1 to sensor *i*, *h*_1_(*k*) represents the trial-to-trial variation of the signal *s*_1_(*j*) of source 1, and likewise *L*_2_(*i*), *h*_2_(*k*), and *s*_2_(*j*). Of course, *L*_1_(*i*) *h*_1_(*k*) *s*_1_(*j*)+*L*_2_(*i*) *h*_2_(*k*) *s*_2_(*j*) is not identical to $\psi_{i}^{\left(k\right)}R_{\text{ij}}$ in (4), thus (4) is an approximation rather than the exact representation. Therefore, the method *formally* applies, i.e. it is able to perfectly disentangle *h*_1_ from *h*_2_, only when the two leadfields are mutually orthogonal, i.e. when *L*_1_(*i*)*L*_2_(*i*) = 0 for all *i*; then, the Hadamard product of the two related forward fields *L*_1_(*i*) *h*_1_(*k*) *s*_1_(*j*) and *L*_2_(*i*) *h*_2_(*k*) *s*_2_(*j*) is a zero field. However, since the two simulated sources were clearly separated in space, we assumed that this was practically the case.

**Figure 1:**
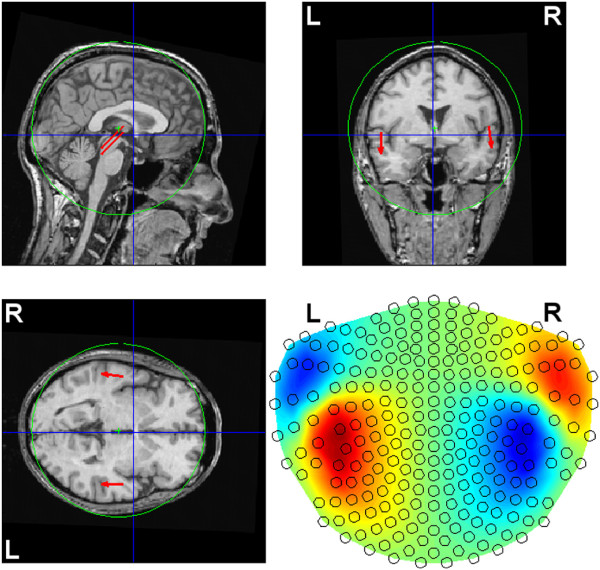
**Simulated ECD locations.** Two tangential ECDs of unit length were seeded (at the back ends of the red arrows) in the auditory cortices. The corresponding forward field is shown in the bottom-right panel. The spherical head model was applied, and the green sphere was fitted using FieldTrip [32], based on 192 surface points selected from head digitization. Only the points close to the two auditory cortices were used for the fit. The blue lines intersect at the anterior commissure.

**Figure 2:**
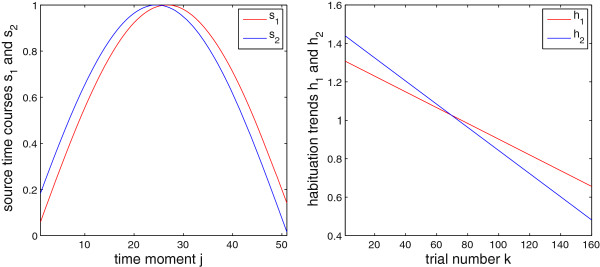
**Simulated ECD time courses.** Left: Time course of the left- (red) and the right-hemisphere source (blue); see figure 1. Right: Simulated habituation trends for the two sources.

Based on a magnetic-resonance-imaging (MRI) scan of our subject (see Section *Material*), we selected one location per auditory cortex where the M100/N100 response (see, e.g., [29-31], and references therein) to simple 1-kHz tones is supposed to be generated. Assuming the spherical head model, at each of these locations we seeded a tangential ECD of unit moment (figure 1), whose amplitude time course was simulated as the [ *x*,*y*,*z*]-moment multiplied by a normalized time series generated as *s*_1_(*j*) = sin(3*j*/*J*) for the left hemisphere and *s*_2_(*j*) = sin(3*j*/*J* + *π*/25) for the right hemisphere, to simulate the M100 wave at its peak proximity and to account for a possible phase (or peak latency) difference between the time courses of the two sources; see figure 2. In the simulation, *J* = 51, which covered 50 ms at the sampling frequency of 1017.25 Hz. These trial-invariant time courses were next multiplied across trials by scalar coefficients originating from two linear trends (one for each of the two ECDs) with negative slopes, to model the habituation of the two sources across trials. Namely, each of the two linear trends was a vector containing *K* weighting coefficients. Those vectors were simulated as *h*_1_(*k*) = -*k* + 2*K* for *s*_1_ and *h*_2_(*k*) = -*k* + 3*K*/2 for *s*_2_, and scaled according to (5) each, resulting, for *K* = 160, in the slopes: -0.0041 for the left and -0.0060 for the right source.

**Figure 3:**
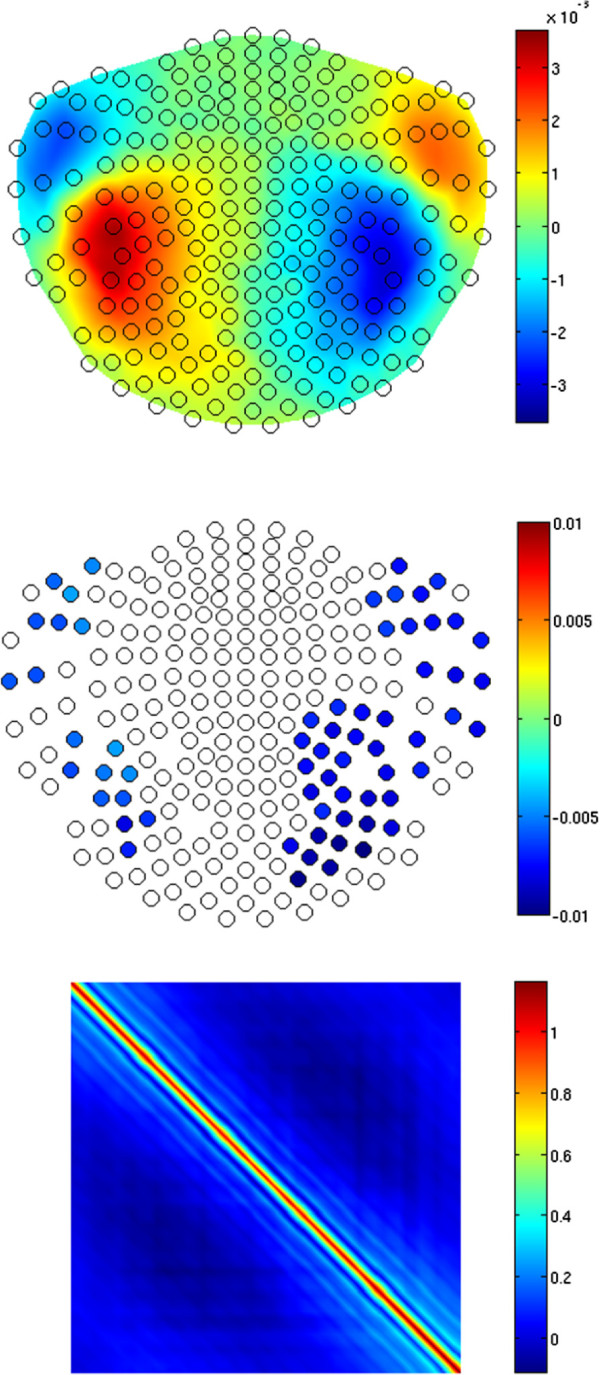
**Results of simulations.** Top: the trial-invariant spatial response pattern *R* at *j* = 25, i.e. for the time point at the middle of the analyzed time window (see also figure 2) in a 2D projection of the MEG sensors layout (open black circles), for the simulated data generated as the superposition of the habituating activity (see figure 2) of the ECDs depicted in figure 1 and the contaminating spatially and temporally correlated background noise, at the overall SNR=0.1. Middle: Spatial distribution of the slope coefficients of 1-st degree polynomials fitted to the multichannel estimates of *ψ* reflecting the trial-to-trial variability of *R*. Sensors marked with blue-filled circles denote the negative slopes significantly different from zero at the 0.05/245 significance level, where 245 is the number of analyzed channels, revealing a clear habituation. The exact color of a circle reflects the value of the slope. Bottom: The estimate of the temporal covariance *T* of the noise.

The forward field resulting from the two sources was calculated for each trial *k* and contaminated by noise taken from the -50–0-ms prestimulus baseline period of individual trials of a real MEG measurement of the same subject, whose MRI was used to seed the simulated ECDs. Both the forward-field signal and the noise were normalized with respect to their norms across all channels *i*, samples *j* and trials *k*, and then the noise was scaled such that the overall signal-to-noise (SNR) ratio equaled 0.1, a very demanding but realistic value, which we based on an estimate of SNR from a real data set.

figure 3 shows the estimates obtained from the iterative solution of equations (13)–(16) after 20 iterations. The top panel shows the estimated trial-invariant pattern of the response, *R*, at *j* = 25, i.e. between the two M100 peaks originating from the two ECDs. A strong correspondence to the forward field of the simulated ECDs (see figure 1) can be seen. Since the estimated *ψ*, which scales *R* across trials (see (4)), is typically noisy, 1st-degree polynomials were fitted (see [11,17,18]) to the *ψ* estimates on each channel in order to reveal the main tendency in the overall trial-to-trial variability. figure 3 (middle) shows the slopes of the fitted polynomials for each channel. We only show the values for those sensors at which the value of the slopes was significantly different from zero at the Bonferroni-corrected significance level of 0.05/245, where 245 is the number of artifact-free channels (we excluded 3 sensors from the 248-channel set of our 4D MEG system due to strong artifacts on those channels; let us remind here that we used a real-measurement noise in these simulations). Sensors marked with empty circles denote the slopes which were not significantly different from zero. Note that those are predominantly channels with poor SNR, as revealed by the top panel. The significant slopes reveal the difference in habituation between the two auditory cortices, with the mean equal to -0.0041 for the left-hemisphere sensors and -0.0056 for the right hemisphere, compared to -0.0041 and -0.0060 at the source level. In both cases, the corresponding standard deviation *σ* was equal to 0.0007. Hence, the habituation lateralization on the source level was well reconstructed on the sensor level despite the demanding overall SNR of 0.1.

The bottom panel of figure 3 shows the estimate of the temporal covariance, *T*, of the contaminating noise, which reveals some nonstationarity [33,34]. We do not show the somewhat peculiar pattern of the estimate of *X*, since it stems from the particular spiral arrangement of the sensors labels in the 4D MEG system, which makes visual inspection of *X* impaired.

**Figure 4:**
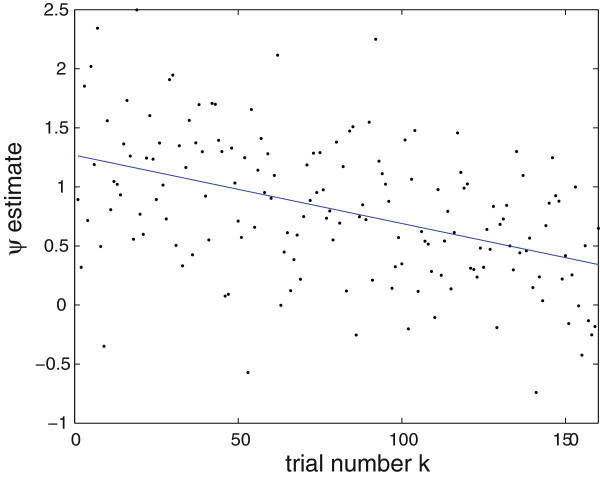
**Habituation.** 1st-degree polynomial (blue line) fitted to *ψ*^(*k*)^ for a right-hemisphere channel revealing a strong signal and a clear habituation (see also figure 3). For this channel, the fitted line *p*(*k*) = -0.0058 *k* + 1.2681.

For illustrative purposes, figure 4 shows, for a selected channel revealing a strong signal and a clear habituation, the estimated *ψ*^(*k*)^ (black dots) and the consecutively fitted 1st-degree polynomial.

**Figure 5:**
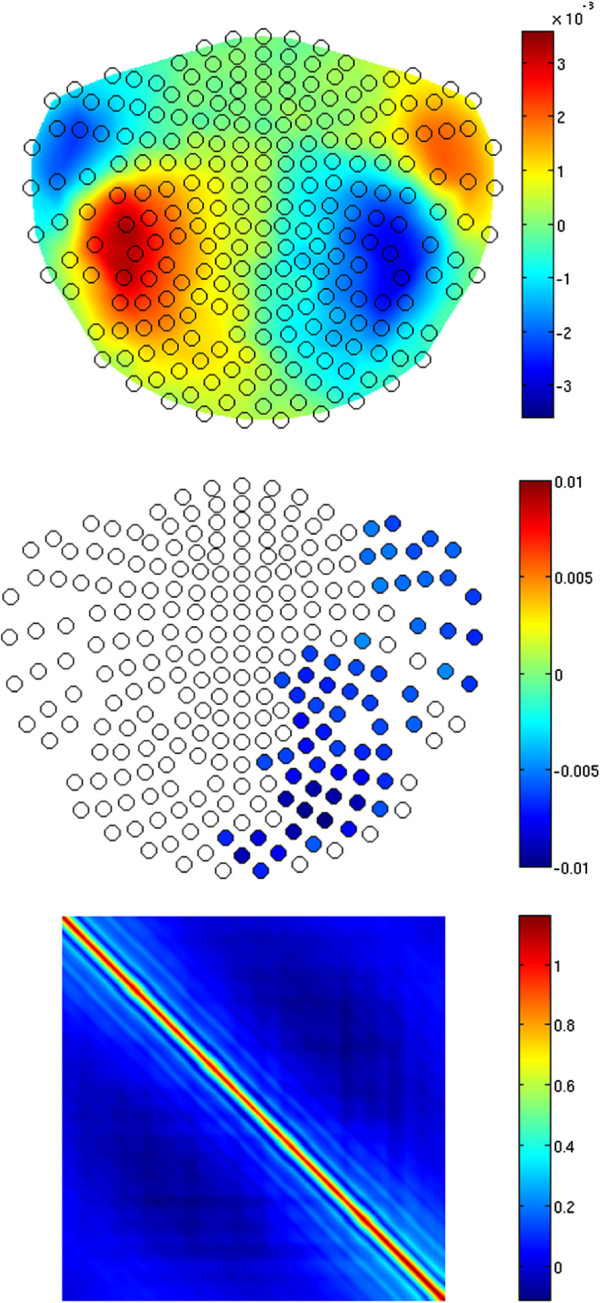
**Results of simulations.** Like in figure 3 but for another simulation in which the left-hemisphere ECD did not habituate. Note the spatial selectivity of the habituation estimate on the sensor level (the middle figure).

In addition, we simulated a scenario in which only one of the two sources, namely the one in the right hemisphere, habituated. For that source, habituation strength was the same as before, whereas for the left-hemisphere EDC, *h*_1_(*k*) = 1 independent of *k*. figure 5 shows results of the estimation, in full analogy to those from figure 3. Note that the algorithm managed to reconstruct the habituation on the sensors that were located above the right hemisphere, while those for the left hemisphere remained essentially unaffected. This time the mean of the significantly non-zero slopes of the 1st-degree polynomials fitted to the estimates of *ψ* for the right-hemisphere sensors was equal to -0.0066 (with *σ* = 0.0012), still close to the slope of the simulated *h*_2_, i.e. to -0.0060.

**Figure 6:**
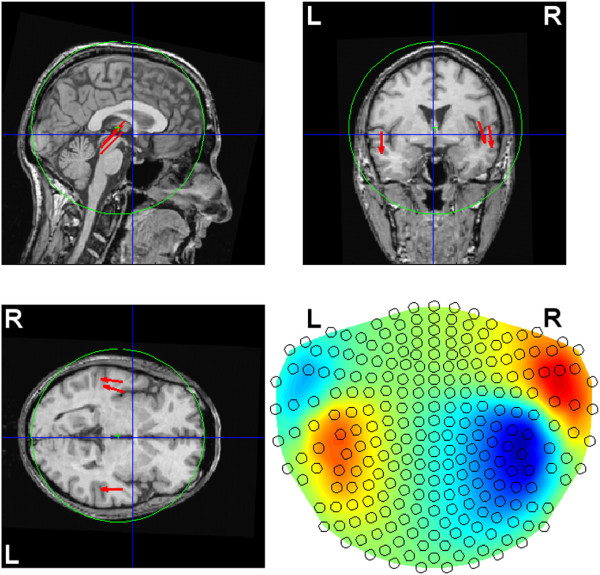
**Simulated ECD locations.** Like in figure 1 but with the additional (third) ECD in the right auditory cortex.

**Figure 7:**
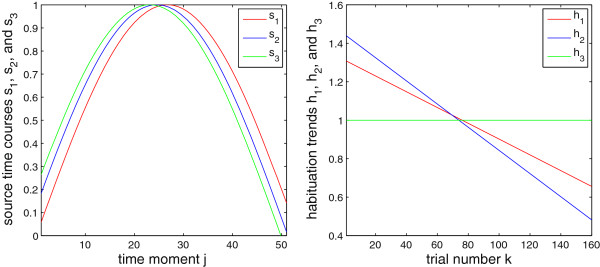
**Simulated ECD time courses.** As in figure 2 but for the three sources shown in figure 6, with the green color corresponding to the additional ECD in the right hemisphere.

**Figure 8:**
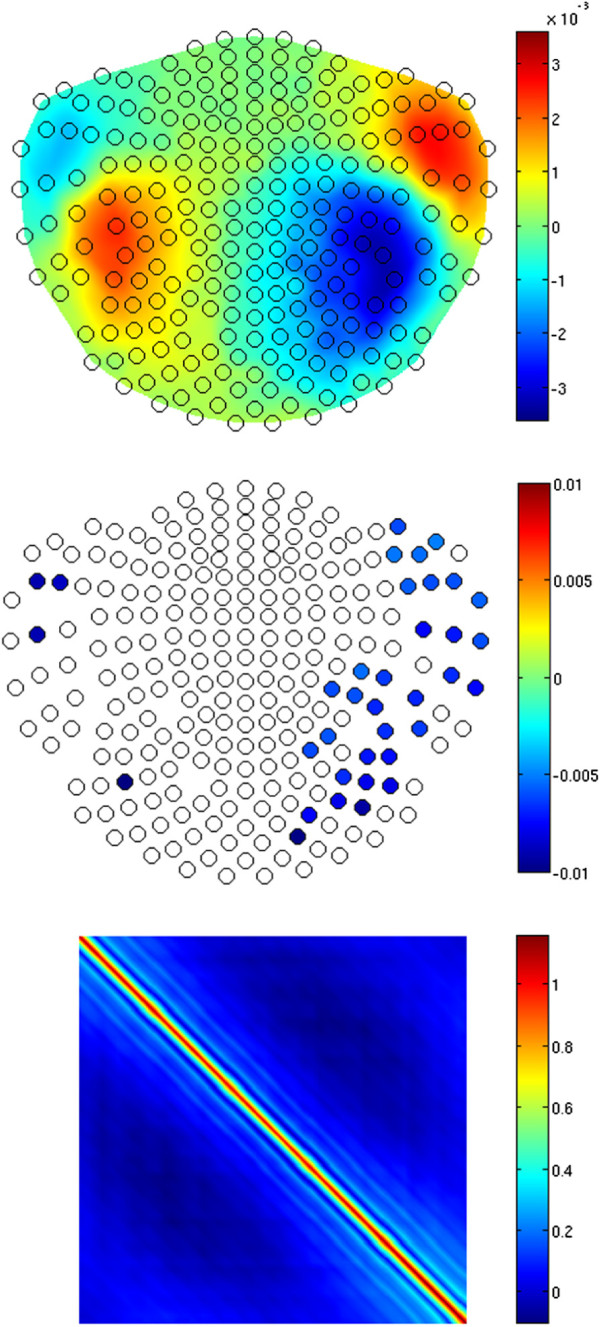
**Results of simulations.** Like in figures 3 and 5 but for the scenario depicted in figures 6 and 7.

In order to test the performance of the method in situations in which the leadfields of the ECDs are far from mutual orthogonality, we simulated an additional scenario in which a third ECD was placed in the right hemisphere; see figures 6 and 7. The time course of this third ECD was simulated as *s*_3_(*j*) = sin(3*j*/*J* + *π*/15) normalized with respect to the peak value. Due to two ECDs in the right hemisphere in this scenario, the overall ECD signal strength in that hemisphere was larger, compared to that in the left hemisphere. Moreover, due to the aforementioned scaling of the signal and the noise resulting in an overall SNR of 0.1, the SNR for the left-hemisphere sensors was now weaker than in the previous simulations. To examine the influence of the trial-to-trial characteristics of the third source on the overall habituation pattern for the right hemisphere, we assumed that this additional source did not habituate, i.e. that its *h*_3_(*k*) = 1 for all *k* (*h*_1_ and *h*_2_ were like in the first simulation; see figure 2). Since, as aforementioned, ECDs whose leadfields are not distinct enough, i.e. not orthogonal or almost orthogonal, can not be efficiently separated, the estimated overall habituation pattern for the right hemisphere is weakened in this scenario; see figure 8. This is due to the fact that the third source did not habituate. The observed pattern is a mixture of the trial-to-trial characteristics of the two sources in the right hemisphere; the mean of the significantly non-zero slopes of the 1st-degree polynomials fitted to the estimates of *ψ* for sensors above the right-hemisphere was equal to -0.0036 (with *σ*=0.0006). This will of course be the case for any real-data scenario with multiple sources located close to each other. However, since the exact number of ECDs is not known in practice, we do not consider such an “integrative”, and hence impaired, resolution of the method a serious flaw. The fact that we do not know, and hence do not want to assume explicitly, the exact number of ECDs for real data is essentially the reason for estimating the trial-to-trial variability on the sensor rather than source level.

Since, as mentioned above, the SNR for the left-hemisphere sensors in this scenario was smaller than in the two previous simulations, the habituation pattern for the ECD of the left auditory cortex was now revealed less clearly. Compared to the result shown in figure 3, the habituation pattern of the left hemisphere is now weaker (although some sensors still reveal significant decay, with the mean significant slope -0.0049, *σ* = 0.0003).

### Real MEG data

**Figure 9:**
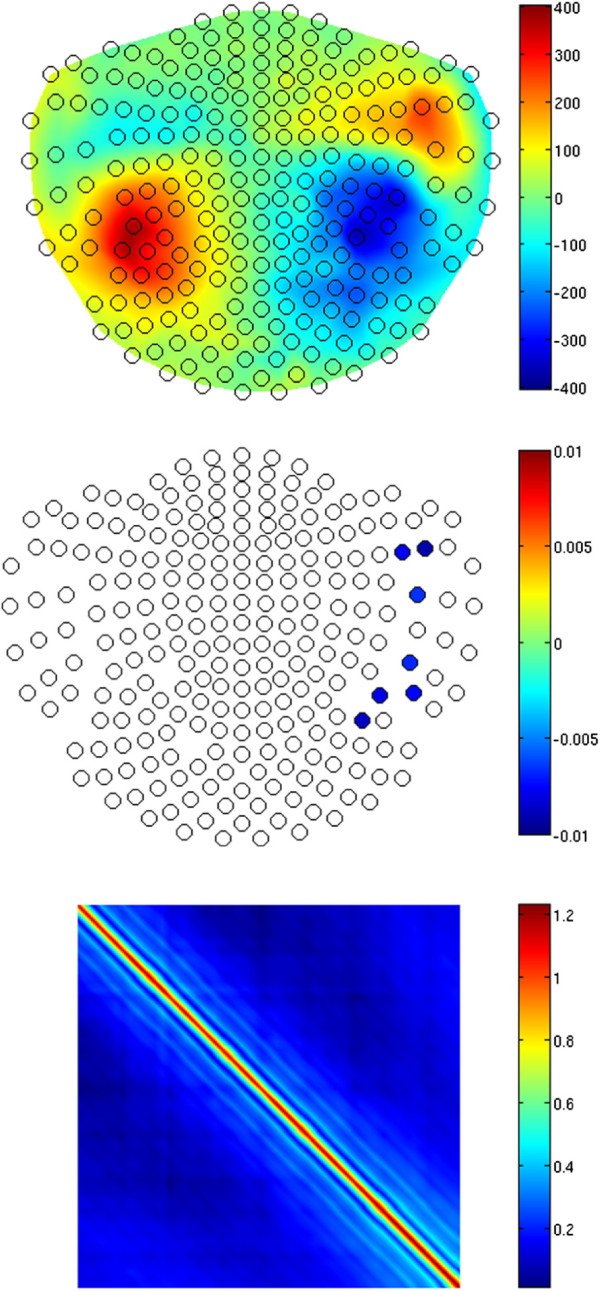
**Results for the real MEG data.** Top: the estimate of *R* [fT] at *j* = 25 corresponding to *t* = 95 ms. Middle: the slopes of 1st-degree polynomials fitted to the estimates of *ψ*. Sensors marked with blue-filled circles reveal a clear habituation at the restrictive 0.05/245 significance level. Bottom: the estimate of the temporal covariance *T* of the noise.

figure 9 shows the findings for real MEG data (see Section *Material*) after 20 iterations (see Section *Calculation of the estimates of ...*). The analyzed time window was 70–120 ms, which covered the positive/negative part of the M100 waveform for the left/right channel with the best SNR.

The top panel of figure 9 shows the estimated trial invariant response *R* at *j* = 25 corresponding to *t* = 95 ms. This pattern looks very close to the average signal at this time point. In the middle panel, a clear habituation pattern is visible for several sensors located above the right auditory cortex, as they reveal significantly negative slopes at the 0.05/245 significance level. The mean of those significant slopes is -0.0041 (*σ* = 0.0005). In this subject, the trial-averaged signal reveals an overall worse SNR above the left, compared to the right hemisphere, which might be the reason for this particular lateralized result.

**Figure 10:**
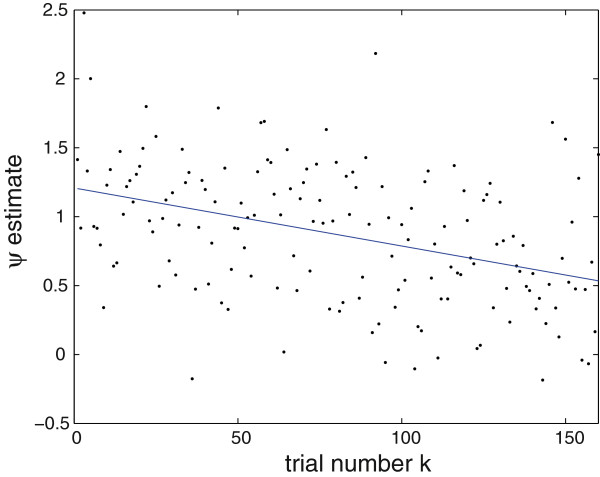
**Habituation in real-data analysis.** The blue line depicts the 1st-degree polynomial *p*(*k*) = -0.0042 *k* + 1.2079 fitted to *ψ*^(*k*)^ for a right-hemisphere channel revealing a strong signal and a clear habituation (see figure 9).

The bottom panel shows the estimated temporal covariance matrix *T* of the noise, which reveals some tiny traces of nonstationarity.

figure 10 for the real-data analysis is fully analogous to figure 4 for the simulation. Alternatively to the 1st-degree polynomial, one might fit an exponential function. However, given the observed level of the inter-trial variance of *ψ* and the fact that in the real-data scenario the exact characteristics of habituation is not known, we decided to follow the Ockham’s razor principle and hence opt for linear fits. Besides, exponential fits resulted in exactly the same channels revealing significant habituation.

## Discussion

We have introduced an extension of the maximum-likelihood approach for the estimation of the trial-to-trial variations of evoked brain responses in MEG based on the work of de Munck *et al.*[11] to obtain sensor-specific estimates. In [11], the trial-invariant spatio-temporal response pattern was weighted in each trial by a scalar coefficient which was identical for all sensors. Here, we further developed this approach by estimating a coefficient for each individual sensor. We checked its applicability using simulated data and found that the lateralization of the habituation simulated on the source level could be reconstructed on the sensor level, even for a very demanding SNR. The analysis of illustrative real MEG data revealed an auditory-cortex related habituation pattern, too, showing that the method is capable of finding significant habituation in data from real experiments.

The estimation of the channel-dependent *ψ* is computationally much more demanding compared to the scenario with *α* assumed identical to all channels [11]. This is so because the dimensionality of the problem increases, while the probability space becomes reduced by one dimension—the number of channels. This makes the overall estimation difficult and possibly less robust compared to the case with the scalar *α*^(*k*)^. Assuming that all the cortical processes were characterized by the same trial-to-trial variability pattern, the use of the simpler approach of de Munck *et al.*[11] would be more appropriate. However, it is not realistic to assume a priori that all processes involved will habituate identically, especially not in scenarios with complex stimulation paradigms. In fact, some may even reveal the opposite behavior, i.e. sensitization (see, e.g., [35]). Even for simple auditory stimuli, the habituation pattern may reveal differences between the two hemispheres—something that could not be addressed with the model of de Munck *et al.*[11]. Hence, in such cases, topographical representations of the trial-to-trial variability will be more appropriate.

An important issue to be taken into consideration when applying the advanced approach presented here is the transition from the source-level model to the sensor-level model. If all sources were characterized by exactly the same trial-to-trial variability, the model with scalar *α*^(*k*)^[11] would be sufficient, as it corresponds to linear scaling of the forward equation in each trial. Such simple scaling is, however, not possible if each source has its own trial-to-trial variation, as assumed in the simulations (see Section *Simulations*). Hence, our model, given by (4), is not exact. We are aware of this inaccuracy, yet, for sources that are sufficiently separated in space, as it is the case when a single ECD per auditory cortex is assumed, this inaccuracy is rather small. In this case, one can treat the problem almost as separate forward problems, one for each source. Of course, the closer in space the sources, the more problematic this issue becomes, which is why the applicability of the proposed approach in scenarios with differently habituating sources in close proximity to each other would be questionable. Alternatively, one could propose a linear expansion into several *α*^(*k*)^*R* products with scalar *α*^(*k*)^s, where each term would correspond to the forward field of a single underlying source. However, this approach would require knowledge of the exact number of sources a priori, something which is usually not available. Therefore, we consider our model to be a reasonable approximation in paradigms where one can assume spatially distinct sources.

We critically examined the convergence of the algorithm and found that the estimates of *ψ* and *R* show some tiny oscillations with consecutive iterations, i.e. that the ratio of the norms of the estimates in two consecutive iterations does not decay monotonically. Nonetheless, in numerous simulations as well as for real data, we found that 20 iterations is a number which, however somewhat arbitrary, results in the spatial distribution of the estimate of *R* resembling that of the average signal very well and in *ψ* nicely revealing the habituation patterns of the underlying sources. A good reason for not proceeding further is—apart from the related time cost—the fact that for real data the algorithm might result in patterns of *R* which would differ from the trial-averaged signal a bit too strongly. This may be caused by some information flow (leakage) between the estimates of *ψ* and *R*. Hence, we infer that about 20 iterations offer a reasonable trade-off between the quality of the estimate of *ψ* and that of the *R* estimate.

The moderate nonstationarity in the estimated temporal covariance of the noise, depicted in figures 3, 5, 8, and 9, may stem from the intrinsic properties of the noise. A recent work by Kipiński *et al.*[34], which examined nonstationarity of MEG data using modern statistical tests adopted from econometrics, revealed variance-nonstationarity of the analyzed signals. However, the nonstationarity of *T* that we observed may also stem from a non-perfect separation of the stochastic noise from the quasi-deterministic evoked response. These considerations correspond to the ongoing debate on the mechanism of the generation of evoked responses and the associated relation of the phase of spontaneous rhythms with respect to the stimulus onset (see, e.g., [36,37], and references therein).

## Endnote

^a^ We do not discuss here the difference between magnetometers or axial and planar gradiometers in this respect. An interested reader is referred to the famous paper of Hämäläinen *et al.*[1].

## Appendix: Detailed derivations

### Estimation of *X* and *T*

Since the likelihood function *f*_*ε*_ attains maximum for the same arguments as its natural logarithm, we can differentiate ln (*f*_*ε*_). We will calculate the derivative of the natural logarithm of (12) with respect to *X*, and set it to zero. Thus, we will differentiate

(18) $$\begin{array}{c} \frac{\partial \ln f_{\varepsilon }}{\mathrm{\partial X}}=-\frac{\partial }{\mathrm{\partial X}}\left(\frac{1}{2}\underset{k}{\sum }\text{tr}\left(\left(R^{\left(k\right)}-\psi^{\left(k\right)}R\right)T^{-1}{\left(R^{\left(k\right)}-\psi^{\left(k\right)}R\right)}^{T}X^{-1}\right)+\right)\\ \left(\;\;\;+\ln\left({\left(2\pi \right)}^{\text{IJK}/2}{\left(\det T\right)}^{\text{IK}/2}{\left(\det X\right)}^{\text{JK}/2}\right)\right)\;. \end{array}$$

Let us notice that for any symmetric, non-singular matrix *X*, it is true (see [27], p. 180) that

(19) $$\frac{\partial }{\mathrm{\partial X}}\det X=\left(\det X\right)X^{-1}\;.$$

Furthermore, since here *X* is square, it is true (see [27], p. 178) that

(20) $$\frac{\partial }{\mathrm{\partial X}}\text{tr}\left(AX^{-1}\right)=-{\left(X^{-1}AX^{-1}\right)}^{T}\;,$$

where

(21) $$A=\left(R^{\left(k\right)}-\psi^{\left(k\right)}R\right)T^{-1}{\left(R^{\left(k\right)}-\psi^{\left(k\right)}R\right)}^{T}\;.$$

Therefore, using basic properties of logarithm and the fact that both *X* and *T* are symmetric, we can write

(22) $$\frac{\partial \ln f_{\varepsilon }}{\mathrm{\partial X}}=\frac{1}{2}\underset{k}{\sum }\left(X^{-1}\left(R^{\left(k\right)}-\psi^{\left(k\right)}R\right)T^{-1}{\left(R^{\left(k\right)}-\psi^{\left(k\right)}R\right)}^{T}X^{-1}\right)-\frac{\text{JK}}{2}X^{-1}\;.$$

After setting the expression above to zero, we obtain (13).

Performing very similar derivation, one obtains (14).

### Estimation of *ψ*^(*k*)^ and *R*

Using the facts (see Table 4 on p. 178 in [27]) that

(23) $$\frac{\partial }{\mathrm{\partial M}}\text{tr}(\text{AM})=A^{T}$$

and

(24) $$\frac{\partial }{\mathrm{\partial M}}\text{tr}(\text{MA}M^{T}B)=B^{T}MA^{T}+\text{BMA}\;,$$

it is straightforward to obtain

(25) $$\begin{array}{c} -\frac{1}{2}\frac{\partial }{\partial \psi^{\left(k^{\prime }\right)}}\underset{k}{\sum }\text{tr}\left(\left(R^{\left(k\right)}-\psi^{\left(k\right)}R\right)T^{-1}{\left(R^{\left(k\right)}-\psi^{\left(k\right)}R\right)}^{T}X^{-1}\right)=\\ \;=-\text{dg}\left(X^{-1}R^{\left(k^{\prime }\right)}T^{-1}R^{T}\right)+\text{dg}\left(X^{-1}\psi^{\left(k^{\prime }\right)}RT^{-1}R^{T}\right)\;, \end{array}$$

where dg is the operator which replaces every square matrix *M* with a new one, consisting of the leading diagonal of *M* and having zeros elsewhere. Then, the following algebraic manipulations lead to equation (15). For the first term of the right-hand side (RHS) of equation (25) we can write

(26) $$\forall i\;\;{\left(\text{dg}\left(X^{-1}R^{\left(k^{\prime }\right)}T^{-1}R^{T}\right)\right)}_{\text{ii}}={\left(\text{diag}\left(X^{-1}R^{\left(k^{\prime }\right)}T^{-1}R^{T}\right)\right)}_{i}\;,$$

where *i* = 1,…,*I*, ()_*i* *i*_ denotes the *i*th element of the leading diagonal of an *I*×*I* matrix, and ()_*i*_ denotes the *i*th element of an *I*×1 column vector.

For the second term of the RHS of equation (25) we can write

(27) $$\forall i\;\;{\left(\text{dg}\left(X^{-1}\psi^{\left(k^{\prime }\right)}RT^{-1}R^{T}\right)\right)}_{\text{ii}}=\underset{t,l,m}{\sum }X_{\text{it}}^{-1}\psi_{t}^{\left(k^{\prime }\right)}R_{\text{tl}}T_{\text{lm}}^{-1}{\left(R^{T}\right)}_{\text{mi}}\;.$$

where *t* = 1,…,*I*, *l* = 1,…,*J*, *m* = 1,…,*J*.

Since the RHS of equation (25) must be set to zero and since it contains diagonal terms only, we have *I* equations, one for each *i*, in which the RHS of equation (26) is equal to the RHS of equation (27).

Next, we can rewrite the RHS of equation (27) as

(28) $$\underset{t}{\sum }X_{\text{it}}^{-1}\left(\underset{l,m}{\sum }R_{\text{tl}}T_{\text{lm}}^{-1}{\left(R^{T}\right)}_{\text{mi}}\right)\psi_{t}^{\left(k^{\prime }\right)}=\underset{t}{\sum }\beta_{\text{it}}\;\psi_{t}^{\left(k^{\prime }\right)}\;,$$

where *β* is an *I*×*I* matrix whose elements are given by

(29) $$\beta_{\text{it}}=X_{\text{it}}^{-1}\underset{l,m}{\sum }R_{\text{tl}}T_{\text{lm}}^{-1}{\left(R^{T}\right)}_{\text{mi}}=X_{\text{it}}^{-1}{\left(RT^{-1}R^{T}\right)}_{\text{ti}}\;.$$

Now, since *T*^-1^ is symmetric, so is *RT*^-1^*R*^T^, and hence we can write

(30) $$\beta_{\text{it}}=X_{\text{it}}^{-1}{\left(RT^{-1}R^{T}\right)}_{\text{it}}={\left(X^{-1}⊙\left(RT^{-1}R^{T}\right)\right)}_{\text{it}}\;.$$

Let us then denote the RHS of equation (26) by *γ*_*i*_, i.e. the *i*th element of a column vector *γ*. Since the RHS of (28) is assumed to be equal to *γ*_*i*_, we can write

(31) $$\underset{t}{\sum }\beta_{\text{it}}\psi_{t}^{\left(k^{\prime }\right)}=\gamma_{i}$$

and therefore, in the matrix notation,

(32) $$\beta \text{diag}\left(\psi^{\left(k^{\prime }\right)}\right)=\gamma \;.$$

Then, of course,

(33) $$\text{diag}\left(\psi^{\left(k^{\prime }\right)}\right)=\beta^{-1}\gamma \;,$$

and hence

(34) $$\psi^{\left(k^{\prime }\right)}=\text{dg}\left(\beta^{-1}\gamma \right)\;,$$

which, given the RHS of (30) for the elements of *β* and the RHS of (26) for the elements of *γ*, yields equation (15).

Very similarly, i.e. using (23) and (24), one easily obtains (16).

## Abbreviations

ECD: Equivalent current dipole; EEG: Electroencephalography; MRI: Magnetic resonance imaging; MEG: Magnetoencephalography; RHS: Right-hand side [of an equation]; SNR: Signal-to-noise ratio; SPN: Signal-plus-noise [model].

## Competing interests

The authors declare that they have no competing interests.

## Authors’ contributions

CS proposed the model and the estimation algorithm, derived the estimators of *X* and *T*, performed most of the analyses, and wrote the paper. PK derived the estimators of *ψ* and *R*, performed some analyses, and contributed substantially to interpretation of data; he also critically revised the model and the manuscript. Both authors read and approved the final manuscript.

## Author’s information

^1^Special Lab Non-invasive Brain Imaging, Leibniz Institute for Neurobiology, Brenneckestr. 6, 39118 Magdeburg, Germany. ^2^Team Normal and Abnormal Motor Control, ICM Brain and Spine Institute, Sorbonne University, Pierre-and-Marie-Curie University (Paris 6), INSERM UMR1127, CNRS UMR7225, Hôpital Pitié Salpêtrière, 47 bd de l’Hôpital, 75013 Paris, France. C. SieluŻycki started working on this project while he was with the Special Lab Non-invasive Brain Imaging, Leibniz Institute for Neurobiology, Magdeburg. He is now with the Team Normal and Abnormal Motor Control, ICM Brain and Spine Institute, Sorbonne University, Pierre-and-Marie-Curie University (Paris 6), INSERM UMR1127, CNRS UMR7225, Paris. ^3^ Department of Biomedical Physics, Institute of Experimental Physics, Faculty of Physics, University of Warsaw, ul. HoŻa 69, 00-681 Warszawa, Poland. P. Kordowski is currently a trainee in the Special Lab Non-invasive Brain Imaging, Leibniz Institute for Neurobiology, Magdeburg.
